# Autoimmune Encephalitis Concomitant with SARS-CoV-2 Infection: Insight from ^18^F-FDG PET Imaging and Neuronal Autoantibodies

**DOI:** 10.2967/jnumed.120.249292

**Published:** 2020-12

**Authors:** Stephan Grimaldi, Stanislas Lagarde, Jean-Robert Harlé, José Boucraut, Eric Guedj

**Affiliations:** 1 Aix Marseille Univ, APHM, Timone Hospital, Department of Neurology and Movement Disorders, Marseille, France; 2 Aix Marseille Univ, APHM, INSERM, INS, Inst Neurosci Syst, Timone Hospital, Epileptology Department, Marseille, France; 3 Aix Marseille Univ, APHM, Timone Hospital, Department of Internal Medicine, Marseille, France; 4 Aix Marseille Univ, APHM, CNRS, INT, Conception Hospital, Immunology Laboratory, Marseille, France; and; 5 Aix Marseille Univ, APHM, CNRS, Centrale Marseille, Institut Fresnel, Timone Hospital, CERIMED, Nuclear Medicine Department, Marseille, France

**Keywords:** autoimmune encephalitis, ^18^F-FDG PET, methylprednisolone, SARS-CoV-2, COVID-19

## Abstract

We report the case of a 72-y-old man with concomitant autoimmune encephalitis and severe acute respiratory syndrome coronavirus 2 (SARS-CoV-2) infection. The patient presented with subacute cerebellar syndrome and myoclonus several days after general infectious symptoms began. **Methods:** Clinical examination, CT, PET, MRI, and autoantibody testing were performed. **Results:** The oropharyngeal swab test was positive for SARS-CoV-2. The brain MRI results were normal. Cerebrospinal fluid testing showed normal cell counts, a negative result on reverse-transcription polymerase chain reaction testing, and no oligoclonal banding. Brain ^18^F-FDG PET showed diffuse cortical hypometabolism associated with putaminal and cerebellum hypermetabolism, compatible with encephalitis and especially cerebellitis. The immunologic study revealed high titers of IgG autoantibodies in serum and cerebrospinal fluid directed against the nuclei of Purkinje cells, striatal neurons, and hippocampal neurons. Whole-body ^18^F-FDG PET and CT scans did not show neoplasia. Treatment with steroids allowed a rapid improvement in symptoms. **Conclusion:** This clinical case argues for a possible relationship between SARS-CoV-2 infection and autoimmune encephalitis and for the use of ^18^F-FDG PET in such a context.

Since late December 2019, there has been a worldwide outbreak of severe acute respiratory syndrome coronavirus 2 (SARS-CoV-2), with substantial morbidity and mortality. Most patients present with fever and respiratory tract symptoms (1). Several neurologic manifestations have been described, mostly acute cerebrovascular disease, impaired consciousness, and muscle injury (2). Cases of Guillain–Barré syndrome (3) and meningitis or encephalitis (4) have also been reported. In this context, the possible impact of PET imaging has been debated (5). Here, we report a case of suspected autoimmune encephalitis concomitant with SARS-CoV-2 infection, corroborated by high titers of antineuronal antibodies, ^18^F-FDG PET imaging, and clinical improvement after immunomodulatory treatment.

## CASE REPORT

A 72-y-old nonsmoking man with a single episode of transient global amnesia 10 y previously presented with progressive diffuse arthralgia and a sore throat (day 0). Two days afterward, his general practitioner started josamycin for 4 d. On day 8, the patient exhibited an episode of fever (38°C–38.5°C) without rhinorrhea, cough, or dyspnea, leading to the prescription of azithromycin. He never experienced anosmia or ageusia. On day 12, chest CT showed peripheral bilateral ground-glass lesions and consolidative opacities suggestive of SARS-CoV-2 infection. The symptoms improved gradually. On day 17, he started to develop bilateral upper-limb action tremor. The next day, the tremor worsened, involving the lower limbs and trunk, making walking and sitting impossible and leading to his admission to the hospital.

Neurologic examination showed a cerebellar syndrome (action tremor, ataxia, dysarthria, and upper-limb dysmetria) associated with spontaneous diffuse myoclonus, mostly affecting the proximal limbs and worsening with movement, also stimulus-sensitive. The remainder of the neurologic examination showed normal findings, including ocular motility and deep tendon reflexes. His body temperature, oxygen saturation, respiratory rate, and lung auscultation were normal. A moderate biologic inflammatory syndrome was present, with increased fibrinogen (7.07 g/L; reference level, 1.90–4.30 g/L) and C-reactive protein (14 mg/L; reference level, 0.0–5.0 mg/L).

The first nasopharyngeal swab test for SARS-CoV-2 was positive (day 18), as was the serology afterward (strongly positive for IgM and IgG). The next swab tests, quantitative reverse-transcription polymerase chain reaction testing of nasopharyngeal or oropharyngeal swabs, were negative (days 21, 22, 26, and 27).

**FIGURE 1.**
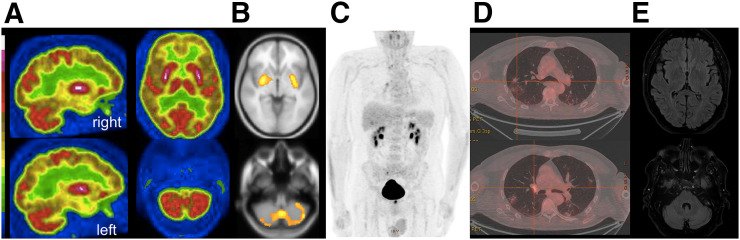
^18^F-FDG brain, whole-body PET, and brain MRI findings. (A and B) Brain ^18^F-FDG PET sagittal and axial slices showing diffuse cortical hypometabolism associated with putaminal and cerebellum hypermetabolism (A), confirmed by SPM comparison to 20 healthy elderly subjects (B) (*P* < 0.001, k > 600). (C and D) Whole-body ^18^F-FDG PET/CT showing slight lung and hilar lymph node hypermetabolism on maximum-intensity projection (C) and axial fused slices (D), matching pseudonodular retractile consolidation postinfectious findings on CT, without straightforward argument for neoplasia. (E) Axial slices from T2-weighted fluid-attenuated inversion recovery MRI of putamen and cerebellum showing no abnormalities.

Electroencephalography showed symmetric diffuse background slowing, reactive to stimulation, without interictal paroxysm and, notably, no correlation of the myoclonus on back-averaging. Brain MRI with gadolinium contrast injection had normal results (Fig. 1). Brain PET with ^18^F-FDG showed putaminal and cerebellum hypermetabolism associated with diffuse cortical hypometabolism, confirmed by whole-brain voxel-based SPM quantification (Fig. 1). Slight lung and hilar lymph node hypermetabolism was noticed, matching postinfectious pseudonodular retractile consolidation on CT, without a straightforward argument for neoplasia.

**FIGURE 2.**
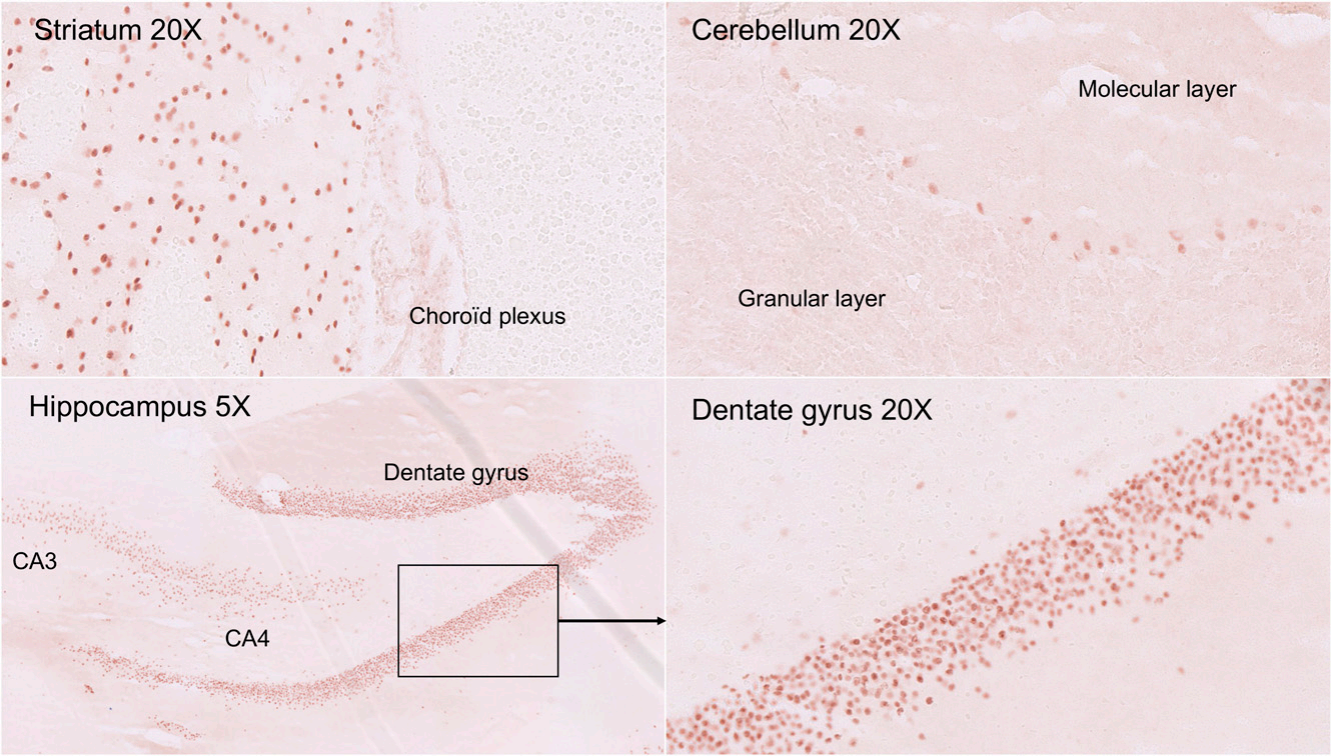
Immunostaining of neuronal nuclei; 25-μm sections were obtained from frozen rat nerve tissue and incubated with different dilutions of serum or CSF and peroxidase-labeled anti-IgG antisera. Immunostaining was revealed in presence of 3-amino-9-ethylcarbazole. This NDP.view2 (Hamamatsu) figure shows labeling with CSF diluted to one sixth on nuclei of Purkinje cells in cerebellum, as well as striatal and hippocampal neurons.

Cerebrospinal fluid (CSF) testing showed normal cell counts (4 × 10^6^/L), a mildly elevated protein level (49 mg/dL), negative reverse-transcription polymerase chain reaction, and no oligoclonal banding. Nerve tissue immunostaining with the serum and CSF revealed the presence of autoantibodies directed against the nuclei of Purkinje cells, striatal neurons, and hippocampal neurons, as illustrated with the CSF in Figure 2. This immunostaining did not evoke any previously described targets for autoimmune encephalitis. The titer of the IgG isotype autoantibodies in the serum was extremely high (1/25,000) but was 1/96 in the CSF. The same intensity and reactivity in serum and CSF were observed at the same IgG concentration, ruling out intrathecal autoantibody synthesis in concordance with the absence of CSF-specific IgG oligoclonal banding. Dot blot assays against onconeural antigens and cell-based assays against membrane antigens had negative results. Finally, the patient was negative for antinuclear, antiphospholipid, and antipolynuclear autoantibodies and other tissue autoantibodies.

Because of the suspected autoimmune nature of the encephalitis, we promptly started immunomodulatory treatment: at first, beginning on day 23, we used intravenous immunoglobulins (0.4 g/kg/d for 5 d); tolerance was good, but there was no significant clinical improvement. We then decided to treat the patient with intravenous methylprednisolone (1 g/d for 5 d) beginning on day 30. To help reduce myoclonus, we introduced oral drops of clonazepam (0.3 mg 3 times a day), but the treatment was stopped because of drowsiness. Clinically, the patient improved considerably after the methylprednisolone treatment, leading to cessation of myoclonus and upper-limb dysmetria and allowing him to walk without assistance. He was discharged from the hospital on day 37. We did not observe any signs of SARS-CoV-2 reactivation.

## DISCUSSION

Like other viruses in the β-coronavirus group, SARS-CoV-2 is suspected to have neuroinvasive potential. The supposition is that a rapid entry into human host cells is mediated mainly by cellular receptor angiotensin-converting enzyme 2, which is expressed in human airway epithelia, vascular endothelia, and the brain, particularly the brain stem (both in neurons and in glia) (6). However, in our patient, a primary infection of the central nervous system seemed unlikely for several reasons. The first was that the neurologic symptoms began at the time that the infectious symptoms were regressing (with only slight residual thoracic abnormalities on PET/CT). The second was the negative results from the next nasopharyngeal or oropharyngeal swab reverse-transcription polymerase chain reaction tests. The third was the absence of a meningeal reaction and the negativity of SARS-CoV-2 reverse-transcription polymerase chain reaction testing in CSF. In contrast, the autoimmune hypothesis was supported by, first, the very high titer of IgG antineuronal autoantibodies, excluding potential induction of antibodies secondary to neuronal damage; second, the brain PET hypermetabolism pattern compatible with encephalitis and especially cerebellitis (7); and third, the clinical improvement after immunomodulatory treatments (8).

It was interesting to note the correlation of clinical symptoms with brain PET hypermetabolism and the targets of the autoantibodies. The mismatch between the abnormalities found on brain PET and the normality of MRI also highlights the potential of PET as an early biomarker (9). Absence of oligoclonal banding or hypercellularity should not preclude the disease, as this absence is frequently reported in other types of autoimmune encephalitis (e.g., contactin-associated proteinlike 2 or leucine-rich glioma inactivated 1 autoantibodies). These types of encephalitis are also characterized by very high titers of autoantibodies, passage of autoantibodies into the central nervous system, and a good response to steroids (10,11). In our patient, antibodies were present at very high levels in the serum and were not synthesized in the central nervous system, probably acting by passage through the CSF (11).

Finally, an alternative hypothesis could be an incidental association between SARV-Cov2 infection and autoimmune encephalitis as, for example, part of a paraneoplastic syndrome. However, no neoplasia was initially detected on whole-body ^18^F-FDG PET and CT scans in this nonsmoking patient, lung findings were compatible with SARS-CoV-2 postinfection lesions (12), and all antionconeural test results were negative. Obviously, follow-up remains necessary to exclude a tumor occurrence.

Because of the presence of antibodies with intracellular targets, methylprednisolone seemed the best first-line treatment option (8). We were at that moment nevertheless concerned about a potential worsening of infectious symptoms due to corticosteroids, and we initially started intravenous immunoglobulins. The recent results of the RECOVERY (Randomised Evaluation of COVID-19 Therapy) trial would now certainly encourage use of steroids as first-line therapy (13). Because of the lack of significant clinical improvement after 5 d of intravenous immunoglobulins, we then prescribed methylprednisolone, leading to major improvement without signs of viral reactivation.

## CONCLUSION

The medical community should be aware of potential post–SARS-Cov-2 autoimmune syndromes that might not appear until several days after resolution of infectious symptoms. In suspected encephalitis, the search for SARS-CoV-2 (notably in the CSF), the detection of antineuronal autoantibodies, and the use of ^18^F-FDG PET—despite the complexity of the logistics and the procedure—could help to determine the diagnosis and the management of patients, especially in MRI-negative cases. Further studies are required to identify the neuronal autoantigen in order to sustain or discard the hypothesis of cross-reactivity with viral antigens.

## DISCLOSURE

No potential conflict of interest relevant to this article was reported.

**KEY POINTS**

**QUESTION:** Can SARS-CoV-2 infection be associated with autoimmune encephalitis?
**PERTINENT FINDINGS:** We report the case of a 72-y-old man presenting with subacute cerebellar syndrome and myoclonus with oropharyngeal swab findings positive for SARS-CoV-2. An autoimmune encephalitis was suspected despite normal results on brain MRI and CSF testing, because brain ^18^F-FDG PET showed a diffuse pattern compatible with encephalitis, and especially cerebellitis, and because there were high titers of autoantibodies directed against the nuclei of Purkinje cells, striatal neurons, and hippocampal neurons.
**IMPLICATIONS FOR PATIENT CARE:** Despite the complexity of the logistics and procedure in this setting, ^18^F-FDG PET could help to determine the diagnosis and management of such patients, especially in MRI-negative cases. Treatment with steroids brought about a rapid improvement in symptoms.

## References

[1] Wang D Hu B Hu C. Clinical characteristics of 138 hospitalized patients with 2019 novel coronavirus-infected pneumonia in Wuhan, China. JAMA. 2020;323:1061–1069. 32031570 10.1001/jama.2020.1585 PMC7042881

[2] Mao L Jin H Wang M. Neurologic manifestations of hospitalized patients with coronavirus disease 2019 in Wuhan, China. JAMA Neurol. 2020;77:683–690. 32275288 10.1001/jamaneurol.2020.1127 PMC7149362

[3] Toscano G Palmerini F Ravaglia S. Guillain–Barré syndrome associated with SARS-CoV-2. N Engl J Med. 2020;382:2574–2576. 32302082 10.1056/NEJMc2009191 PMC7182017

[4] Moriguchi T Harii N Goto J. A first case of meningitis/encephalitis associated with SARS-coronavirus-2. Int J Infect Dis. 2020;94:55–58. 32251791 10.1016/j.ijid.2020.03.062 PMC7195378

[5] Guedj E Verger A Cammilleri S. PET imaging of COVID-19: the target and the number. Eur J Nucl Med Mol Imaging. 2020;47:1636–1637. 32303786 10.1007/s00259-020-04820-z PMC7163172

[6] Steardo L Steardo L Jr Zorec R Verkhratsky A. Neuroinfection may contribute to pathophysiology and clinical manifestations of COVID-19. Acta Physiol (Oxf). 2020;229:e13473. 32223077 10.1111/apha.13473 PMC7228251

[7] Baumgartner A Rauer S Mader I Meyer PT. Cerebral FDG-PET and MRI findings in autoimmune limbic encephalitis: correlation with autoantibody types. J Neurol. 2013;260:2744–2753. 23900756 10.1007/s00415-013-7048-2

[8] Macher S Zimprich F De Simoni D Höftberger R Rommer PS. Management of autoimmune encephalitis: an observational monocentric study of 38 patients. Front Immunol. 2018;9:2708. 30524441 10.3389/fimmu.2018.02708 PMC6262885

[9] Solnes LB Jones KM Rowe SP. Diagnostic value of ^18^F-FDG PET/CT versus MRI in the setting of antibody-specific autoimmune encephalitis. J Nucl Med. 2017;58:1307–1313. 28209905 10.2967/jnumed.116.184333 PMC6944181

[10] Jarius S Hoffmann L Clover L Vincent A Voltz R. CSF findings in patients with voltage gated potassium channel antibody associated limbic encephalitis. J Neurol Sci. 2008;268:74–77. 18068189 10.1016/j.jns.2007.11.004

[11] van Sonderen A Ariño H Petit-Pedrol M. The clinical spectrum of Caspr2 antibody–associated disease. Neurology. 2016;87:521–528. 27371488 10.1212/WNL.0000000000002917 PMC4970662

[12] Setti L Kirienko M Dalto SC Bonacina M Bombardieri E. FDG-PET/CT findings highly suspicious for COVID-19 in an Italian case series of asymptomatic patients. Eur J Nucl Med Mol Imaging. 2020;47:1649–1656. 32342191 10.1007/s00259-020-04819-6 PMC7184819

[13] Oxford University news release low-cost dexamethasone reduces death by up to one third in hospitalised patients with severe respiratory complications of COVID-19. Randomised Evaluation of COVID-19 Therapy website. https://www.recoverytrial.net/files/recovery_dexamethasone_statement_160620_v2final.pdf. Published June 16, 2020. Accessed October 6, 2020.

